# Comparative Biology Research on the Reproductive Organs Between *Physalis pubescens* L. and *Solanum lycopersicum* var. *cerasiforme*

**DOI:** 10.3390/plants15162524

**Published:** 2026-08-20

**Authors:** Xuemeng Shan, Xuechao Feng, Lida Zhang, Lingxia Zhao

**Affiliations:** 1 Department of Plant Science, School of Agriculture and Biology, Shanghai Jiao Tong University, Shanghai 200240, China; shanxuemeng@sjtu.edu.cn (X.S.); mycjcaq@126.com (X.F.); zhangld@sjtu.edu.cn (L.Z.); 2 Joint Tomato Research Institute, School of Agriculture and Biology, Shanghai Jiao Tong University, Shanghai 200240, China

**Keywords:** inflated sepal, fruit development, anther connective tissue, sepal development, microspore development, *MADS-box* genes, comparative biology

## Abstract

The post-anthesis sepal inflation forming a Chinese lantern in *Physalis pubescens* represents a striking morphological novelty, whereas in the related genus *Solanum lycopersicum* var. *cerasiforme*, the sepals remain non-enclosing, providing an ideal comparative system to study reproductive organ divergence. This study aimed to systematically compare the reproductive development between *Physalis pubescens* L. and *Solanum lycopersicum* var. *cerasiforme*. We integrated phylogenetic analysis, light and scanning electron microscopy, semi-thin sectioning, and quantitative RT-qPCR analysis of six *MADS-box* genes. Phylogenetic analysis placed *P. pubescens* in a clade with *P. alkekengi* (L.) and *P. ixocarpa* (Brot. ex Hornem.), distinct from tomato. Floral organs differed markedly in petal color, anther morphology, and dehiscence type. Microspore development was delayed before the tetrad stage but accelerated thereafter in *P. pubescens*; *S. lycopersicum* anthers exhibited pronounced connective tissue proliferation absent in *P. pubescens*. *P. pubescens* sepals expanded 6.92-fold by 17 days post anthesis and enclosed the fruit, while *S. lycopersicum* sepals grew minimally. Sepal expansion was driven by inner epidermal cell enlargement and intercellular space formation, producing a hollow structure with a trichome-free inner epidermis. Expression of six *MADS-box* genes exhibited diversity: *PfMPF2* and *PfAGL1* were upregulated during sepal growth, *PfMPF3*, *PfSEP1* and *PfSEP3* exhibited a high–low–high pattern with minima at peak growth, and *PfAGL6* declined. These findings reveal that coordinated epidermal cell expansion, parenchyma cavity formation, and a dynamic *MADS-box* gene network govern the inflated sepal syndrome, and highlight key divergences in anther morphogenesis, providing a cellular and molecular framework for reproductive evolution in *Solanaceae*.

## 1. Introduction

*Physalis pubescens* L. (downy groundcherry), native to South America, is a semi-cultivated fruit crop now grown in many temperate and subtropical regions, and has become an economically important fruit in Northeast China [1,2]. The plant produces globose yellow berries prized for their sweet–tart flavor and rich nutritional value. Consistent with its traditional medicinal use, extracts and isolated compounds from *Physalis* species have demonstrated diverse pharmacological activities. For example, *physalin* E, a secosteroid purified from *Physalis angulata* L., inhibited lipopolysaccharide (LPS)-induced nuclear factor-κB (NF-κB) activation in RAW 264.7 macrophages at concentrations of 10–30 μM in vitro [3], supporting the anti-inflammatory properties attributed to the genus. The most striking feature of *P. pubescens* is the post-anthesis behavior of its sepal. After pollination, the sepals undergo dramatic enlargement to form a papery, lantern-like structure that completely encloses the fruit, a phenomenon termed inflated sepal syndrome [4,5]. The inflated sepal syndrome serves as a physical barrier against herbivory and mechanical damage and has evolved in multiple solanaceous genera [6,7,8]. In sharp contrast, the sepal of tomato (*S. lycopersicum*), a close relative and model solanaceous plant, remains small and non-enclosed throughout development, providing an ideal comparative system [9,10,11]. Beyond sepal differences, the two species diverge in multiple reproductive traits: for example, *P. pubescens* has pale yellow petals with a purple eye-like pattern and purple anthers with fertile apical necks and dorsal dehiscence, whereas *S. lycopersicum* has yellow lanceolate petals and apically sterile, ventrally dehiscent anthers. These species also differ in microspore developmental timing, anther connective tissue proliferation, and fruit maturation patterns [12,13]. The temporal shift in microspore development—delayed before meiosis but accelerated after tetrad release in *Physalis*—may reflect divergent regulation of conserved developmental programs, such as the *AMS* and *MYB*-dependent networks controlling pollen differentiation [14]. In contrast, the dramatic connective tissue proliferation observed in *S. lycopersicum* anthers, which is completely absent in *P. pubescens*, has been linked to the formation of the anther cone and may represent an evolutionary adaptation to buzz-pollination [15]. However, comparative analyses integrating these traits remain unexplored.

The inflated sepal syndrome is regulated by a network of MADS (MCM1, AGAMOUS, DEFICIENS, and SRF)-box transcription factors [16,17,18]. Heterotopic expression of *PfMPF2* in the sepal is the key trigger of inflated sepal syndrome [4]. Mutations in the *PfMPF2* promoter abolish repression by the A-class protein MPF3, enabling sepal-specific expression [19]. PfMPF2 interacts with SEPALLATA proteins Pf-SEP1, Pf-SEP3, and PfAGL6 to form regulatory complexes that presumably activate downstream genes involved in cell division and cell wall remodeling, thereby driving the rapid post-anthesis calyx expansion [20]. In *S. lycopersicum*, the orthologs of these genes show markedly different expression patterns and the corresponding proteins may not assemble into functionally equivalent complexes, failing to induce sepal inflation [17,21,22,23]. Despite these advances, how the expression of positive and negative regulators is coordinated to control the timing and magnitude of sepal expansion, and how broader reproductive traits differ between these two species, remain poorly understood.

However, the cellular basis of sepal inflation and the coordinated regulation of *MADS-box genes* during this process remain poorly understood. Moreover, how anther developmental programs differ between *P. pubescens* and *S. lycopersicum* has not been systematically characterized. The aim of this study was to elucidate the cellular and molecular basis of sepal inflation and to identify key divergences in reproductive development between *P. pubescens* and *S. lycopersicum*. To this end, we integrated phylogenetic analysis, morphological characterization of floral organs, fruits and microspore development, semi-thin sectioning, scanning electron microscopy, and quantitative expression analysis of six key *MADS-box* genes throughout reproductive development. Our results establish the cellular and molecular framework for reproductive organs between *P. pubescens* and *S. lycopersicum* and reveal novel divergences in reproductive development between these two *solanaceous* species.

## 2. Results

### 2.1. Phylogenetic Analysis of P. pubescens Within the Solanaceae

**Figure 1:**
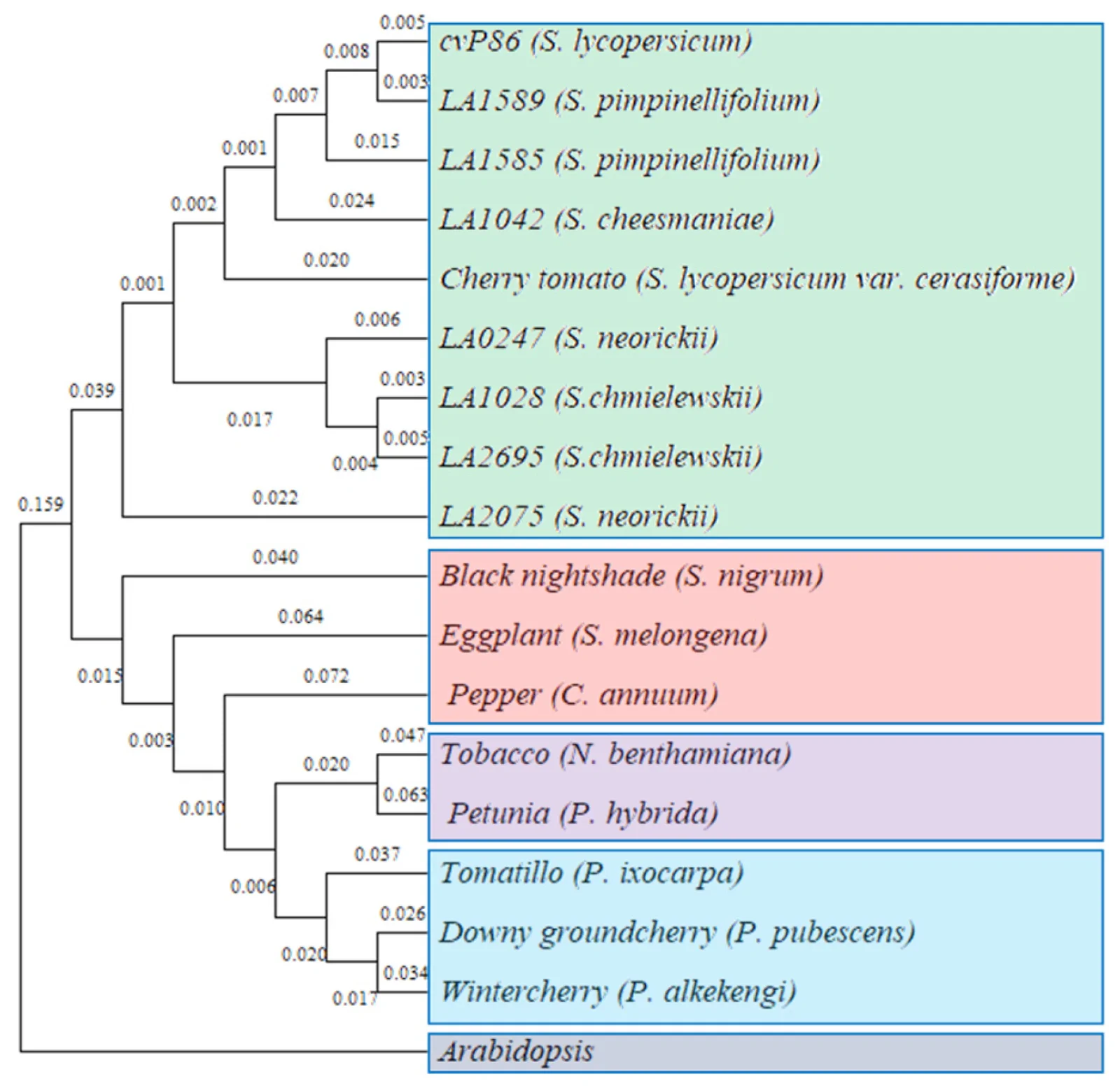
The phylogenetic tree based on *nrDNA-ITS*, *GBSSI* and *trnL-F*. The *P. pubescens* subgroup was marked with a blue frame; the tomato subgroup was marked with a green frame; the *S. nigrum* was marked with a pink frame; the *N. benthamiana* was marked with a purple frame; The *Arabidopsis* was marked with a gray frame. Bootstrap values (1000 replicates) are indicated at the nodes.

To determine the phylogenetic position of *P. pubescens* within the *Solanaceae* and to provide a genetic framework for understanding its unique sepal development, we performed phylogenetic analysis based on three widely used DNA markers: the *Nuclear ribosome deoxyribonucleic acid internal transcribed spacer* (*nrDNA-ITS*), the *Granule-bound starch synthase* (*GBSSI*), and the *Transfer ribonucleic acid leucine-transfer ribonucleic acid phenylalanine* (*trnL-F*). A total of 18 accessions representing 14 species from five genera of *Solanaceae*, together with *Arabidopsis thaliana* (L.) as an outgroup, were included in this study. Genomic DNA was extracted and the target DNA fragments were amplified by PCR using specific primers (Supplementary Figure S1). Genetic distances among the 18 accessions were calculated based on the combined sequence data of the three markers. The results showed that *P. pubescens* had the smallest genetic distance to *P. ixocarpa* and *P. alkekengi*, indicating the closest relationship within the genus, and the highest genetic distance to *A. thaliana*, confirming its distant relationship to the outgroup. Phylogenetic trees constructed using the neighbor-joining method consistently placed *P. pubescens* in a well-supported clade with *P. ixocarpa* and *P. alkekengi*, clearly separated from the tomato subgroup and other subgroups (Figure 1). This phylogenetic distance provides a meaningful evolutionary framework for the comparative analysis of sepal development between *P. pubescens* and tomato.

### 2.2. Plant and Flower Morphology of P. pubescens and S. lycopersicum

**Figure 2:**
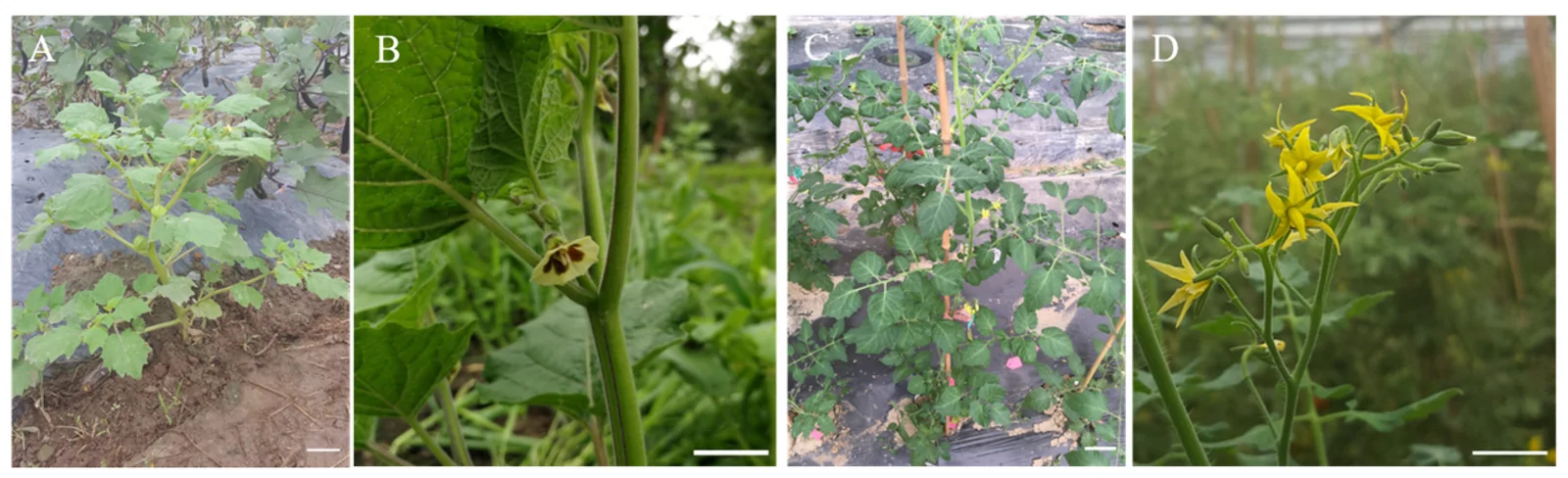
Plant and flower of *P. pubescens* and *S. lycopersicum*. (**A**): The plant of *P. pubescens*, bars = 3 cm; (**B**): the flower of *P. pubescens*, bars = 1 cm; (**C**): the plant of *S. lycopersicum*, bars = 3 cm; (**D**): the flower of *S. lycopersicum*, bars = 1 cm.

To characterize the morphological differences between *P. pubescens* and *S. lycopersicum*, we compared their overall plant architecture, flower, pollen staining and pollen shape. *P. pubescens* exhibited a semi-trailing, determinate growth habit with simple ovate leaves, whereas *S. lycopersicum* displayed an indeterminate, sprawling architecture with compound pinnate leaves (Figure 2A,C). The flowers of *P. pubescens* were smaller than those of *S. lycopersicum*, with pale yellow petals forming a pentagonal corolla that bore a purple eye-like pattern at the throat. In contrast, *S. lycopersicum* cerasiforme produced yellow, lanceolate petals without pigmentation (Figure 2B,D).

**Figure 3:**
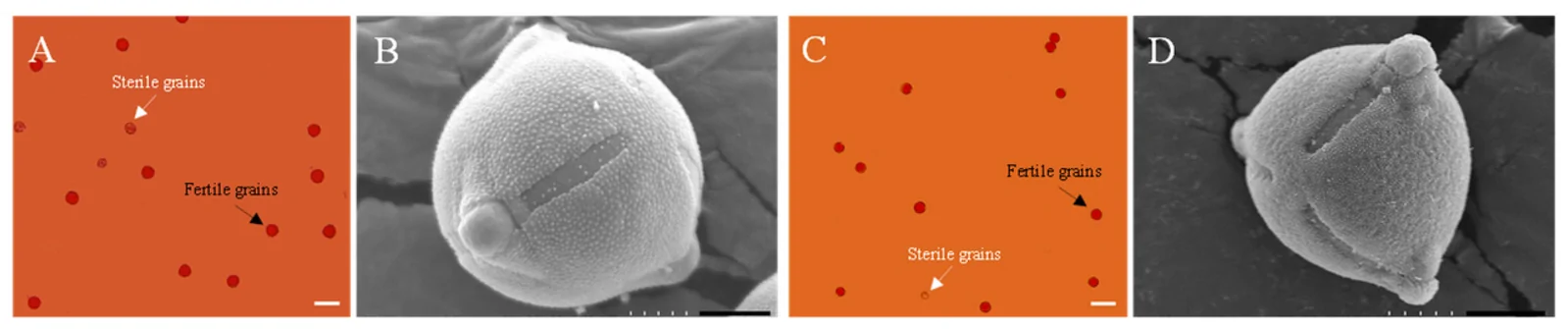
The pollen staining and pollen shape of *P. pubescens* and *S. lycopersicum*. (**A**): Acetocarmine-stained pollen of *P. pubescens*. Fertile grains (black arrows) are round and deeply stained; sterile grains (white arrows) are shrunken and stain weakly, bars = 50 μm; (**B**): the pollen of *P. pubescens*, bars = 5 μm; (**C**): acetocarmine-stained pollen of *S. lycopersicum*. Fertile grains (black arrows) are round and deeply stained; sterile grains (white arrows) are shrunken and stain weakly, bars = 50 μm; (**D**): the pollen of *S. lycopersicum*, bars = 5 μm.

Acetocarmine staining showed that *P. pubescens* and *S. lycopersicum* pollen had high fertility (Figure 3A,C, Supplementary Figure S2). Pollen grains of both species were spherical; however, *P. pubescens* pollen was slightly larger in diameter than that of *S. lycopersicum* (Figure 3B,D).

### 2.3. Floral Organ Morphology and Microspore Development in P. pubescens and S. lycopersicum

**Figure 4:**
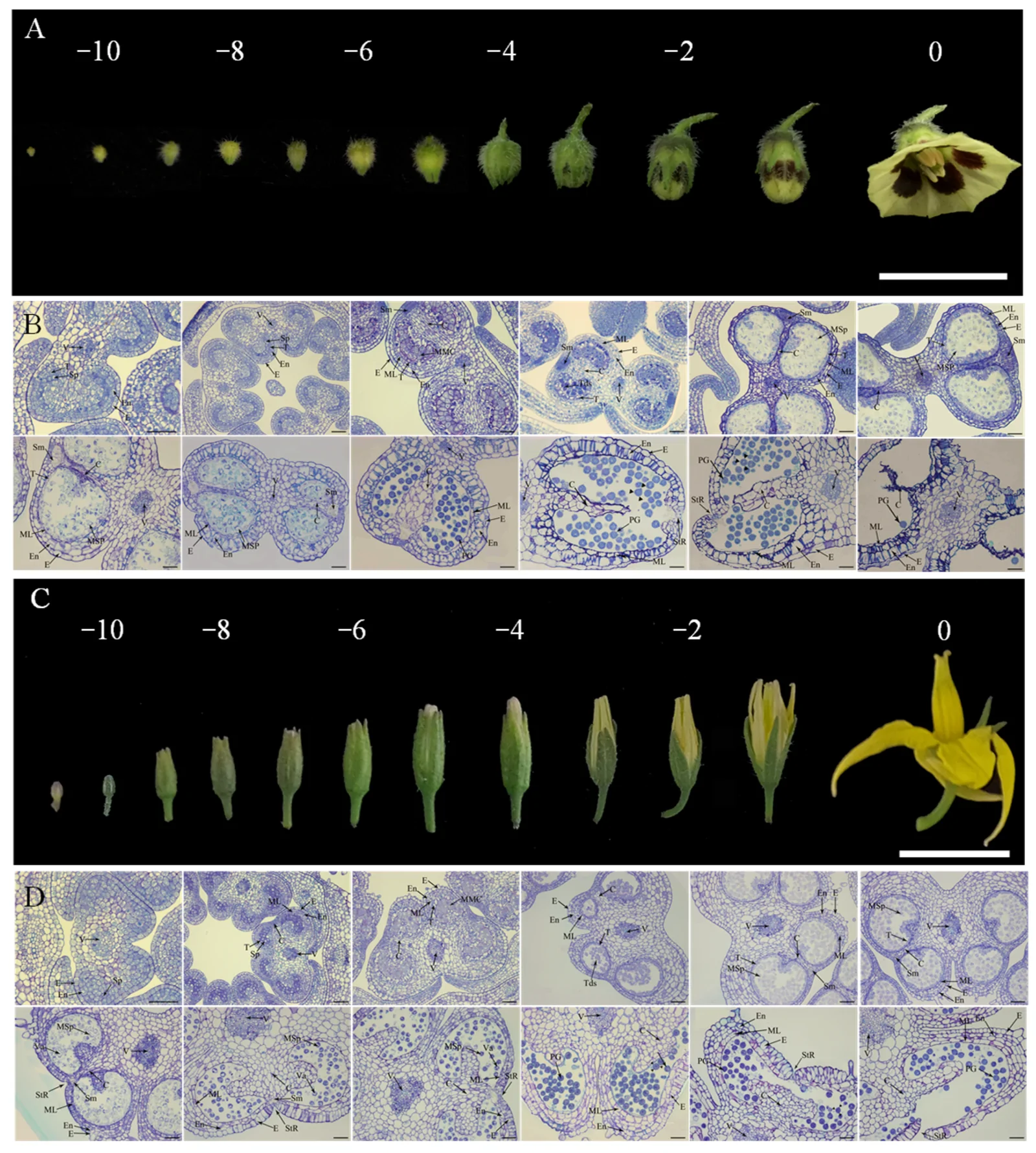
Floral bud development and microsporogenesis in *P. pubescens* and *S. lycopersicum* from −11 dpa to 0 dpa. (**A**): The flower bud development of *P. pubescens*, bars = 1.0 cm; (**B**): semi-thin sections of anther development in *P. pubescens* (−11 dpa, −10 dpa, −9 dpa, −8 dpa, −7 dpa, −6 dpa, −5 dpa, −4 dpa, −3 dpa, −2 dpa, −1 dpa, 0 dpa); bars = 50 μm. At −11 dpa, sporogenous cells were well organized and the tapetum was distinguishable; tetrads appear at −8 dpa; microspores are released from tetrads by −7 dpa and subsequently mature into pollen grains without connective tissue proliferation. (**C**): The flower bud development of *S. lycopersicum*, bars = 1.0 cm; (**D**): semi-thin sections of anther development in *S. lycopersicum* (−11 dpa, −10 dpa, −9 dpa, −8 dpa, −7 dpa, −6 dpa, −5 dpa, −4 dpa, −3 dpa, −2 dpa, −1 dpa, 0 dpa); bars = 50 μm. At −11 dpa, the locules were not yet U-shaped and the tapetum was indistinct; tetrads were already present at −9 dpa; pronounced connective tissue proliferation begins at −7 dpa and progressively occupies more than half of the locule volume by anthesis (0 dpa). C, connective tissue; E, epidermis; En, endothecium; ML, middle layer; MMC, microspore mother cells; MSp, microspores; PG, pollen grains; Sm, septum; Sp, sporogenous cells; StR, stomium region; T, tapetum; Tds, tetrads; V, vascular bundle; Va, vacuoles.

We further compared floral bud development from −11 dpa to anthesis (0 dpa) in the two species. At −11 dpa, buds of both species were yellowish and tightly closed, but their subsequent developmental diverged distinctly. *P. pubescens* buds remained yellow and tightly closed until approximately −5 dpa, whereas buds of *S. lycopersicum* began to turn green at −10 dpa and started to open at approximately −9 dpa. At anthesis, the pale-yellow petals of *P. pubescens* displayed a characteristic purple eye-like pattern at the throat, and the pale purple anthers possessed fertile apical necks and dehisced via dorsal sutures. In contrast, *S. lycopersicum* had yellow, lanceolate petals without pigmentation patterning, and its yellow anthers were apically sterile, laterally connate, forming an anther cone, and dehisced ventrally (Figure 4A,C).

Semi-thin transverse sections of anthers revealed that microspore development in both species could be divided into eleven distinct stages (Figure 4B,D). At the U-shaped sporogenous cell stage (−11 dpa), a single layer of sporogenous cells was already well organized in *P. pubescens*, whereas in *S. lycopersicum*, the four locules had not yet adopted a U-shaped configuration and the tapetum was indistinguishable. Before the tetrad stage (−10 to −9 dpa), microspore development in *P. pubescens* was delayed relative to *S. lycopersicum*: at −9 dpa, *S. lycopersicum* had already entered the tetrad stage, whereas *P. pubescens* was still at the microspore mother cell stage. After the release of uninucleate microspores from tetrads (after −8 dpa), however, development in *P. pubescens* gradually overtook that in *S. lycopersicum*, and pollen maturation and shedding occurred slightly earlier. The most striking cellular divergence was the abnormal proliferation of the connective tissue between the two locules of a single theca in *S. lycopersicum*, which began at −7 dpa, progressively protruded into the locular cavity, and occupied more than half of the locule volume at anthesis, a feature that is completely absent in *P. pubescens* (Figure 4). Collectively, these results demonstrate that the two *solanaceous* species show distinct developmental programs during anther morphogenesis.

### 2.4. Comparative Morphology and Post Anthesis Sepal Development in P. pubescens and S. lycopersicum

**Figure 5:**
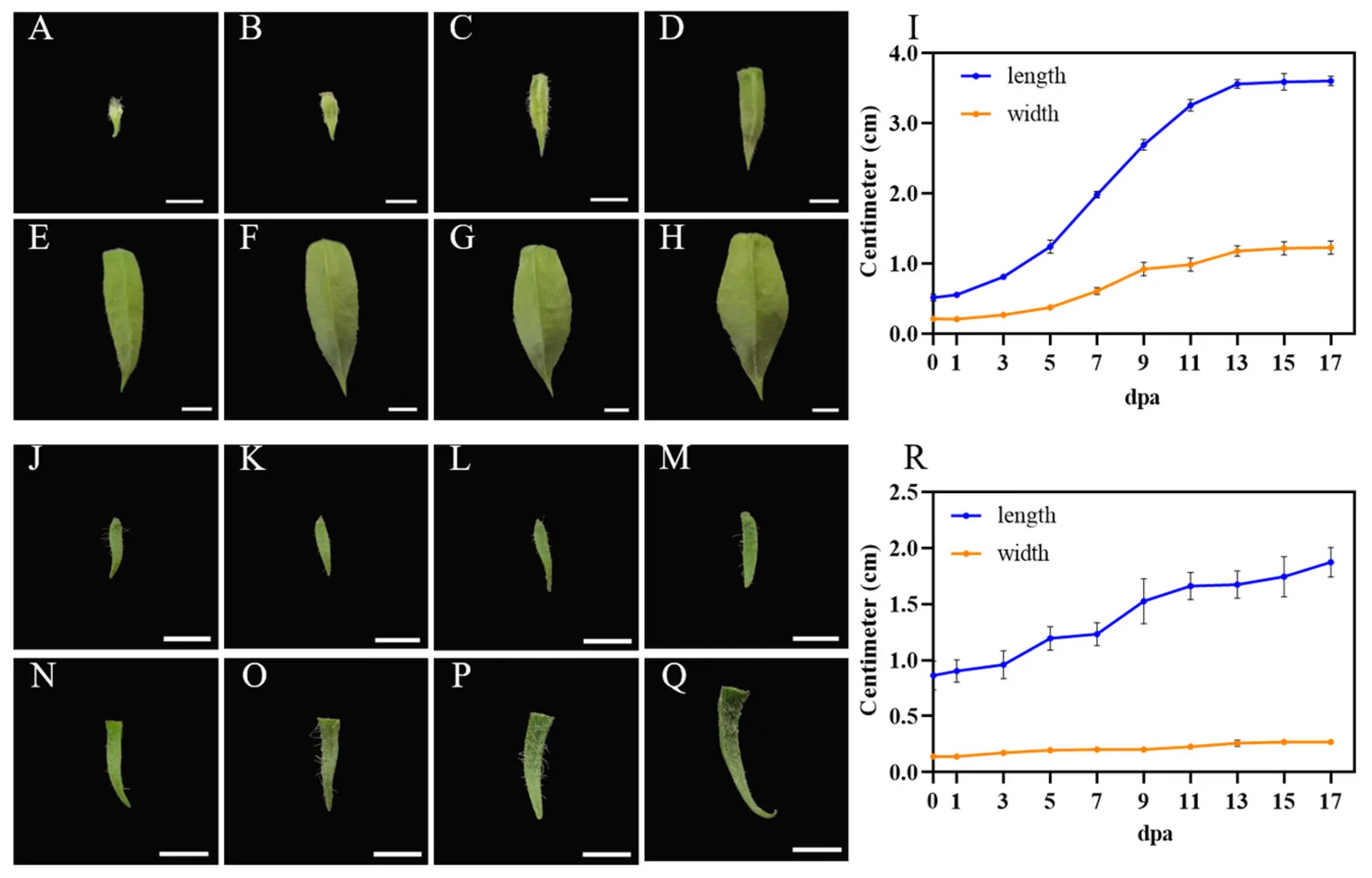
Comparative sepal morphology and growth dynamics of *P. pubescens* and *S. lycopersicum*. (**A**–**H**): The sepal development (1 dpa, 3 dpa, 5 dpa, 7 dpa, 9 dpa 11 dpa, 13 dpa, 15 dpa) of *P. pubescens*, bars = 5 mm; (**I**): the sepal length and width of *P. pubescens*; (**J**–**Q**): the sepal development (1 dpa, 3 dpa, 5 dpa, 7 dpa, 9 dpa 11 dpa, 13 dpa, 15 dpa) of *S. lycopersicum*, bars = 5 mm; (**R**): the sepal length and width of *S. lycopersicum*.

To characterize the differences in sepal development between *P. pubescens* and *S. lycopersicum*, we compared their sepal development morphology and sepal growth dynamics after anthesis. The post anthesis developmental patterns of the sepal were markedly different between *P. pubescens* and *S. lycopersicum*. The sepal of *P. pubescens* grew rapidly, while the *S. lycopersicum* grew relatively slowly (Figure 5I,R). In addition, the midrib of the *P. pubescens* sepal protruded to form prominent ridges, while it was not obvious in *S. lycopersicum* (Figure 5A–H,J–Q). At anthesis (0 dpa), the sepal of *P. pubescens* consisted of five sepals with a bell-shaped structure, which was 0.52 ± 0.05 cm in length and 0.21 ± 0.01 cm in width. The sepal of *S. lycopersicum* was deeply split, with a length of 0.86 ± 0.13 cm and a width of 0.14 ± 0.01 cm (Figure 5I,R). After pollination, the sepal of *P. pubescens* expanded rapidly, and the vein area protruded to form obvious ridges, and finally developed into a completely closed, lantern-shaped structure, which wrapped the whole developing fruit. In contrast, the growth of *S. lycopersicum* sepal after flowering was very limited, and only a small inverted structure remained, which adhered to the basal end of the fruit and did not form a package. To precisely quantify the differential growth patterns, we measured sepal length and width at 2-day intervals from 1 dpa to 17 dpa. The sepal of *P. pubescens* displayed a pronounced sigmoidal growth curve, characterized by a relatively slow initial phase (1–5 dpa), followed by a rapid exponential growth phase (5–13 dpa), and finally reaching a plateau (13–17 dpa). By 17 dpa, the *P. pubescens* sepal had attained a length of 3.60 ± 0.07 cm and a width of 1.23 ± 0.09 cm, representing a 6.92-fold increase in length and a 5.86-fold increase in width relative to their values at anthesis. In contrast, the *S. lycopersicum* sepal exhibited only marginal growth throughout the same period, reaching a final length of 1.87 ± 0.13 cm and width of 0.27 ± 0.01 cm at 17 dpa, corresponding to merely 2.17-fold and 1.93-fold increases, respectively.

These results demonstrate that the formation of the Chinese lantern phenotype in *P. pubescens* is primarily driven by a phase of accelerated sepal expansion initiated around 5–7 dpa, a developmental program that was largely absent in the sepal of *S. lycopersicum*. This pronounced difference in post-anthesis growth trajectory provided a quantitative framework for investigating the underlying cellular and molecular mechanisms of inflated sepal syndrome.

### 2.5. The Morphological Comparison of Different Fruit Development Stages in P. pubescens and S. lycopersicum

**Figure 6:**
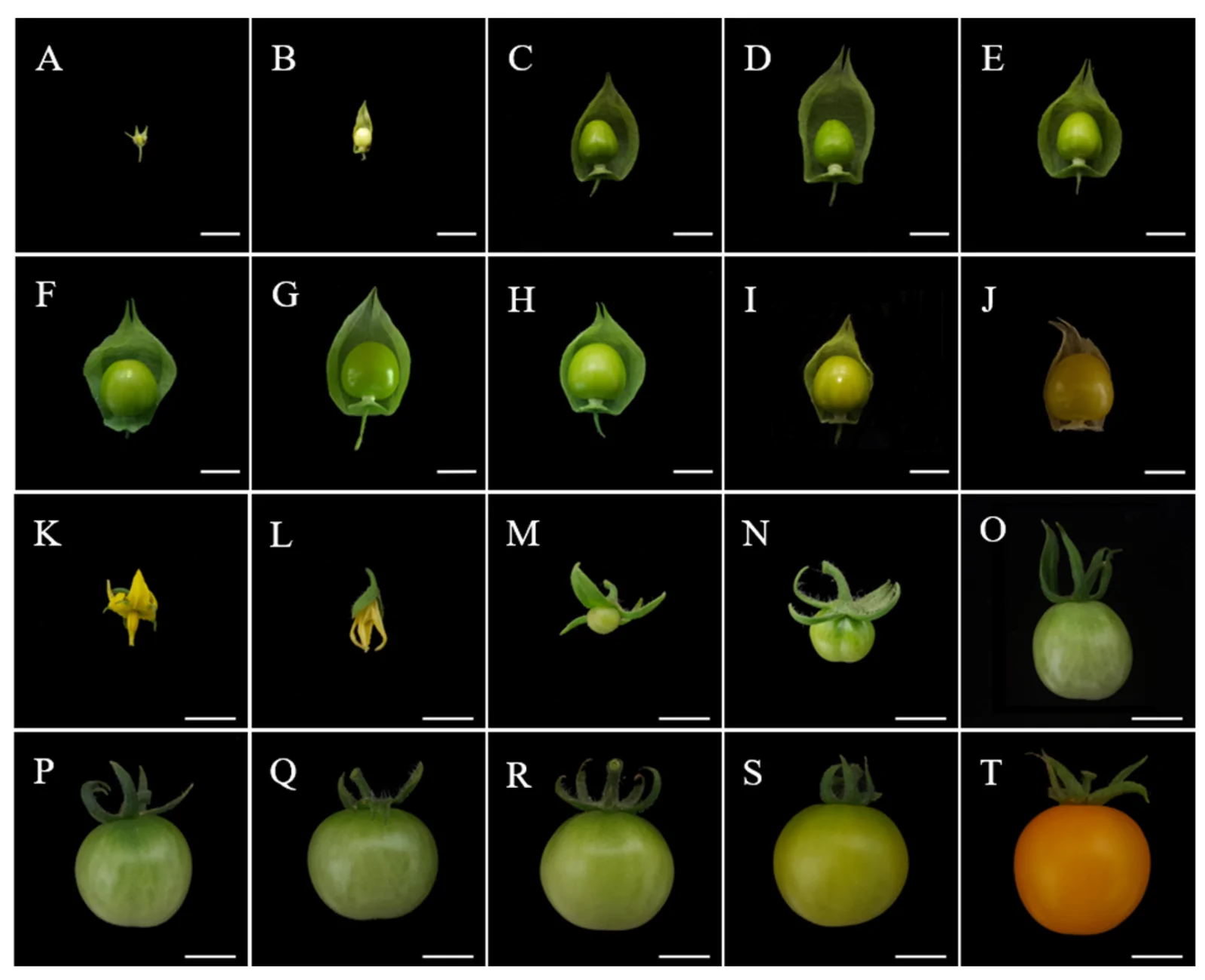
The morphological changes during fruit development and ripening in *P. pubescens* and *S. lycopersicum*. (**A**–**J**): The developmental stages (1 dpa, 5 dpa, 9 dpa, 13 dpa, 17 dpa, 21 dpa, 23 dpa, 25 dpa, break ripen stage, ripen stage) of *P. pubescens* from anthesis to full maturity; (**K**–**T**): the developmental stages (1 dpa, 5 dpa, 9 dpa, 13 dpa, 17 dpa, 21 dpa, 23 dpa, 25 dpa, break ripen stage, ripen stage) of *S. lycopersicum* from anthesis to full maturity; bars = 1.0 cm.

The fruit development of *P. pubescens* is structurally characterized by the coordinated growth of the accrescent sepal and the inner berry. Following anthesis and initial fruit set, the sepal rapidly closes and undergoes a dramatic, lantern-like expansion synchronized with berry enlargement, completely enveloping the developing fruit. Throughout the developmental trajectory, the berry transitions from an early whitish-green to a uniform bright green, eventually turning a bright golden yellow at full maturity; simultaneously, the outer sepal gradually de-greens, desiccates, and ultimately transforms into a brown, paper-like husk (Figure 6A–J).

Distinct from *P. pubescens*, *S. lycopersicum* fruit development exhibits naked berry growth accompanied by substantial volume expansion. Following petal senescence, abscission, and successful fruit set, the young fruit emerges from the basal persistent green sepals and enters a rapid enlargement phase, gradually shaping into a smooth, plump, subglobose form. The pericarp maintains a dark or light green coloration throughout the immature green stages until entering the breaker stage upon ripening initiation, transitioning through yellowish-green before ultimately shifting into a uniform, highly saturated bright orange at the fully ripe stage (Figure 6K–T).

### 2.6. The Difference in Tissue Structure of Sepal in Cross Section

**Figure 7:**
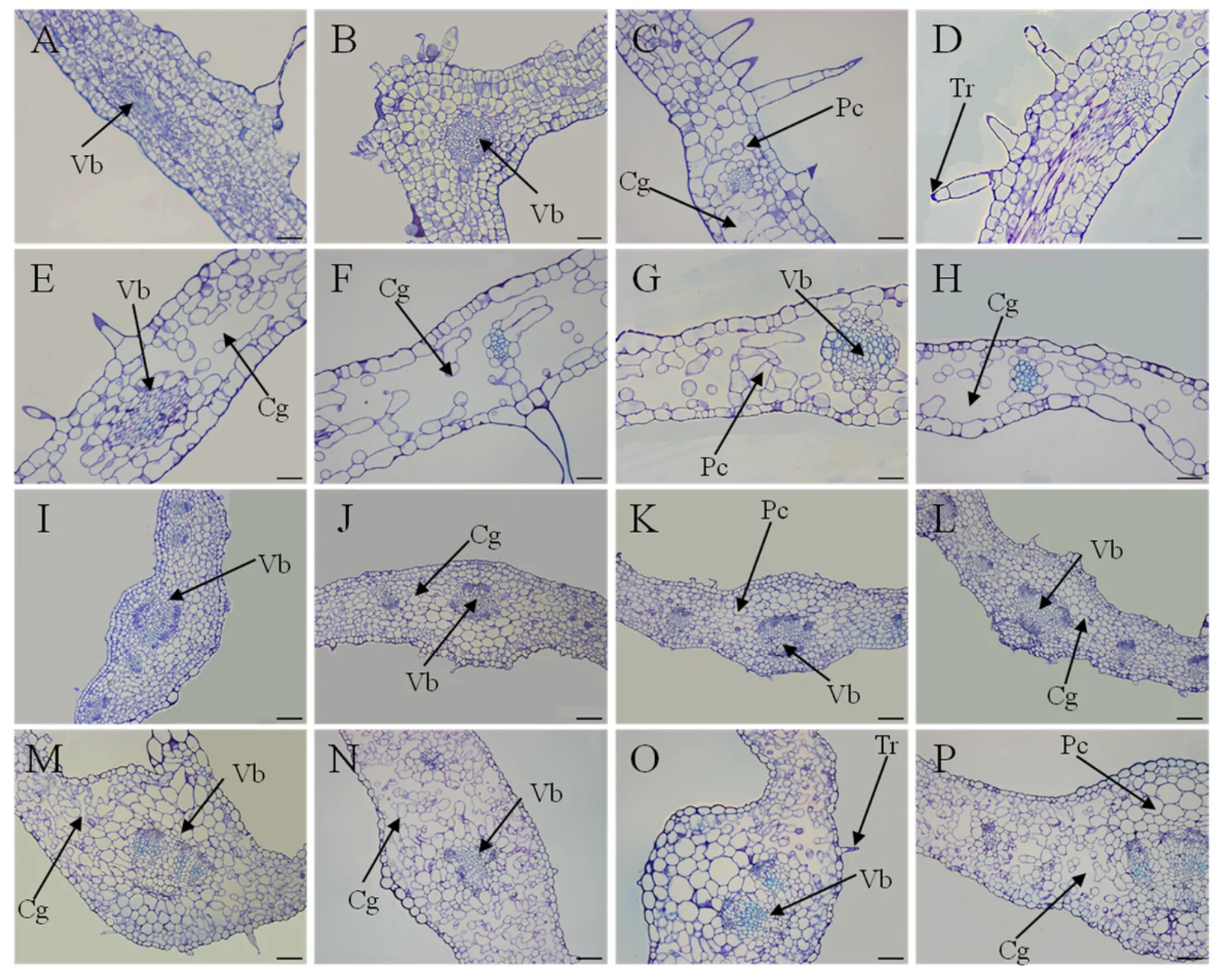
Semi-thin sections of sepal cells in *P. pubescens* and *S. lycopersicum.* (**A**–**H**): Semi-thin sections of sepal cells of *P. pubescens* in sepal development of 1 dpa, 3 dpa, 5 dpa, 7 dpa, 9 dpa 11 dpa, 13 dpa,15 dpa ((**A**): 1 dpa, (**B**): 3 dpa, (**C**): 5 dpa, (**D**): 7 dpa, (**E**): 9 dpa, (**F**): 11 dpa, (**G**): 13 dpa, (**H**): 15 dpa); (**I**–**P**): semi-thin sections of sepal cells of *S. lycopersicum* in sepal development of 1 dpa, 3 dpa, 5 dpa, 7 dpa, 9 dpa 11 dpa, 13 dpa,15 dpa ((**I**): 1 dpa, (**J**): 3 dpa, (**K**): 5 dpa, (**L**): 7 dpa, (**M**): 9 dpa, (**N**): 11 dpa, (**O**): 13 dpa, (**P**): 15 dpa), bars = 100 μm. Semi-thin sections of sepals stained with toluidine blue O. Cg, cell gap; Pc, parenchyma cells; Tr, trichome; Vb, vascular bundle.

To further investigate the microstructure difference between *P. pubescens* and *S. lycopersicum* sepal, we conducted a semi-thin section experiment. The results showed that the sepal of *P. pubescens* and *S. lycopersicum* were composed of inner and outer epidermis, trichomes, parenchyma cells and vascular bundles; however, the epidermis structure was not observed in the inner epidermis of *P. pubescens* (Figure 7). The number of sepal cells in *P. pubescens* was less than that of *S. lycopersicum*, and the size of parenchyma cells increased from 3 dpa, accompanied by a marked increase in intercellular spaces, and by 15 dpa, the parenchyma tissue had become largely hollow. However, the parenchyma cells of *S. lycopersicum* were arranged closely, and the parenchyma cells grow rapidly from 9 dpa, and intercellular spaces only became evident at this stage. Different from other tissues dyed light blue, vascular bundle tissues were dyed with toluidine blue to form small and dense oval tissues. The inner and outer sepals of the two species were composed of monolayer cells, which are arranged neatly and interconnected tightly.

### 2.7. The Difference in Epidermal Cells of Sepal

**Figure 8:**
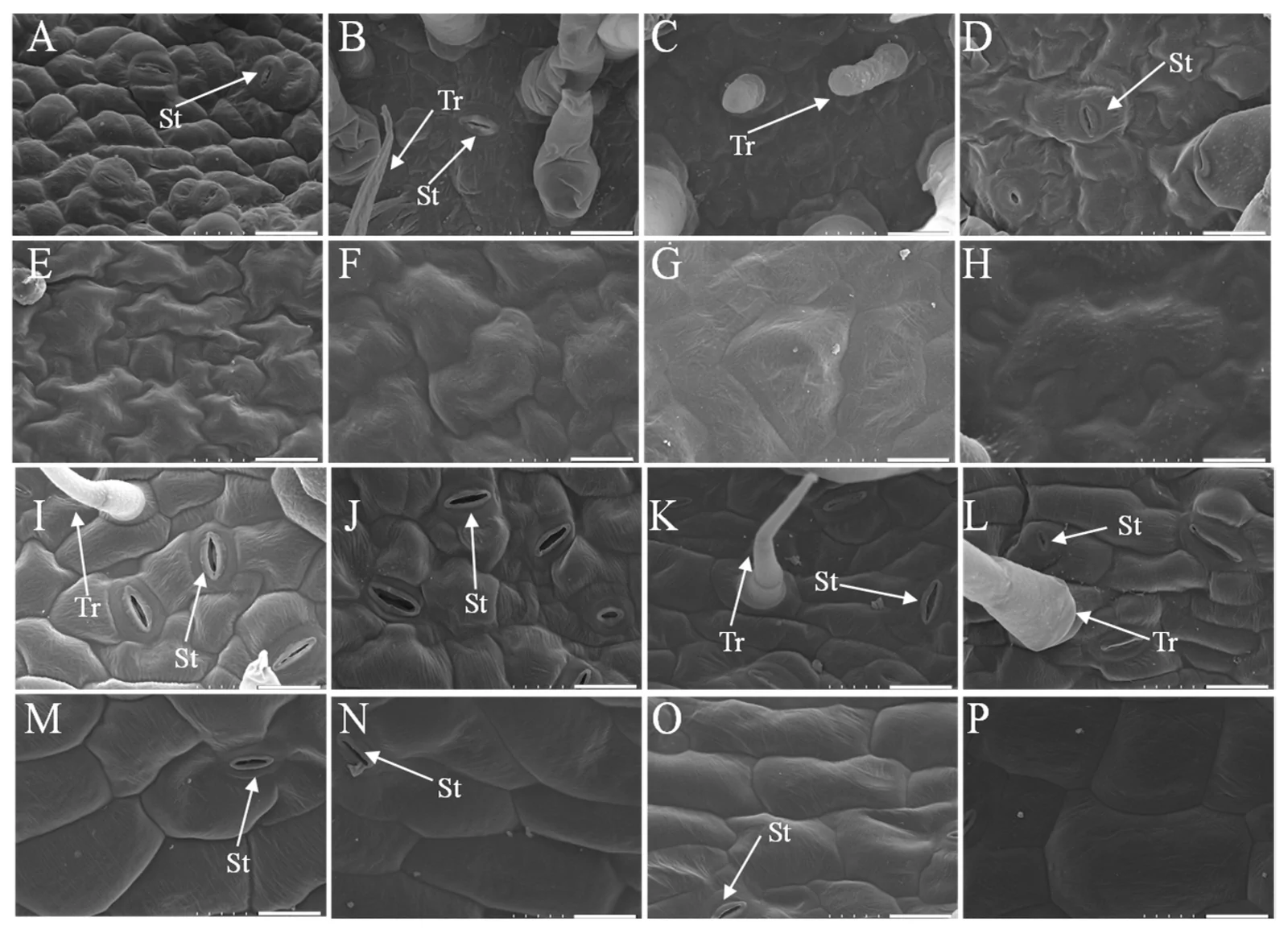
The cell profiles of sepal outer epidermis of *P. pubescens* and *S. lycopersicum*. (**A**–**H**): The cell profiles of sepal outer epidermis of *P. pubescens* in sepal development of 1 dpa, 3 dpa, 5 dpa, 7 dpa, 9 dpa 11 dpa, 13 dpa, 15 dpa ((**A**): 1 dpa, (**B**): 3 dpa, (**C**): 5 dpa, (**D**): 7 dpa, (**E**): 9 dpa, (**F**): 11 dpa, (**G**): 13 dpa, (**H**): 15 dpa); (**I**–**P**): the cell profiles of sepal outer epidermis of *S. lycopersicum* in sepal development of 1 dpa, 3 dpa, 5 dpa, 7 dpa, 9 dpa, 11 dpa, 13 dpa, 15 dpa ((**I**): 1 dpa, (**J**): 3 dpa, (**K**): 5 dpa, (**L**): 7 dpa, (**M**): 9 dpa, (**N**): 11 dpa, (**O**): 13 dpa, (**P**): 15 dpa), bars = 25 μm. St, stomata; Tr, trichome.

Based on the results of the semi-thin section, we conducted a scanning electron microscope. The results showed that there was no difference between the outer epidermal cells of *P. pubescens* sepals and *S. lycopersicum*, and they all have trichomes and a stomatal structure. At 1 dpa, the outer epidermal cells of *P. pubescens* were smaller than those of *S. lycopersicum*. However, by 7 dpa, the outer epidermal cells of *P. pubescens* sepals grew faster than those of *S. lycopersicum*. From the 11 dpa, the outer epidermal cells of *P. pubescens* sepals became larger than those of *S. lycopersicum*, with the cell walls becoming thinner and the cell shape transitioning from oval to irregular (Figure 8).

**Figure 9:**
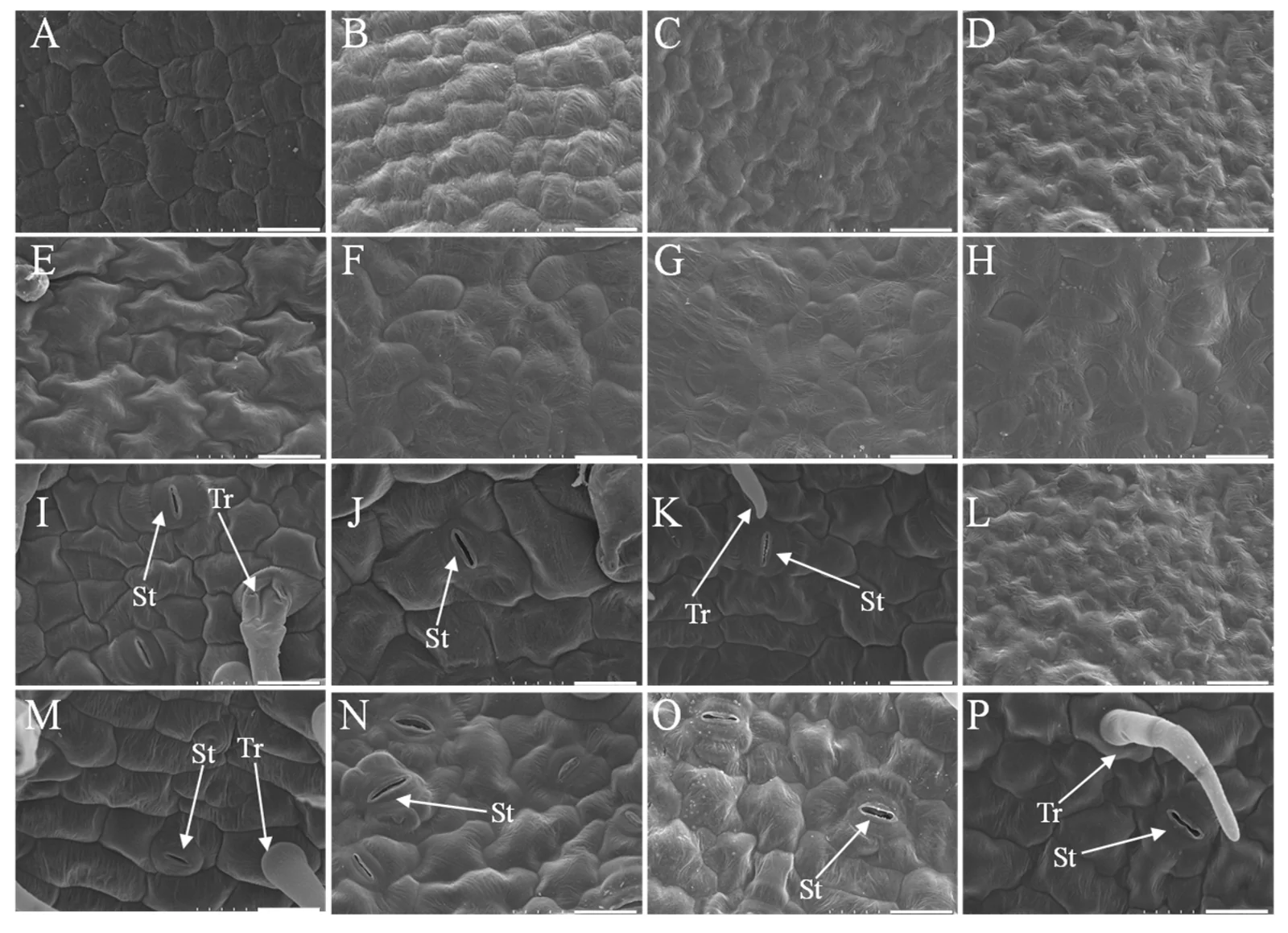
The cell profiles of sepal inner epidermis of *P. pubescens* and *S. lycopersicum*. (**A**–**H**): The cell profiles of sepal outer epidermis of *P. pubescens* in sepal development of 1 dpa, 3 dpa, 5 dpa, 7 dpa, 9 dpa 11 dpa, 13 dpa, 15 dpa ((**A**): 1 dpa, (**B**): 3 dpa, (**C**): 5 dpa, (**D**): 7 dpa, (**E**): 9 dpa, (**F**): 11 dpa, (**G**): 13 dpa, (**H**): 15 dpa); (**I**–**P**): the cell profiles of sepal outer epidermis of *S. lycopersicum* in sepal development of 1 dpa, 3 dpa, 5 dpa, 7 dpa, 9 dpa, 11 dpa, 13 dpa, 15 dpa ((**I**): 1 dpa, (**J**): 3 dpa, (**K**): 5 dpa, (**L**): 7 dpa, (**M**): 9 dpa, (**N**): 11 dpa, (**O**): 13 dpa, (**P**): 15 dpa), bars = 25 μm. St, stomata; Tr, trichome.

The inner epidermis of *S. lycopersicum* sepals was similar to its own outer epidermis with a stomata and epidermis structure, but the epidermis of *P. pubescens* sepals was not observed (Figure 9). The size of epidermal cells in *P. pubescens* was smaller than that of *S. lycopersicum* at 1 dpa. The inner epidermal cells are slightly larger. However, at 7 dpa, the epidermis of *P. pubescens* sepals, especially the inner epidermis, grew faster than that of *S. lycopersicum* (Table 1). From the 11 dpa, the inner and outer epidermis cells of *P. pubescens* sepals were larger than those of *S. lycopersicum*, and the cell wall became thinner and the cell shape became irregular from oval (Figure 8 and Figure 9). The microstructure suggested that the rapid expansion of sepal of *P. pubescens* was mainly caused by the increase in epidermal cell size and parenchyma cell gap of sepals.

### 2.8. Expression Patterns of Genes Related to Sepal Development

**Figure 10:**
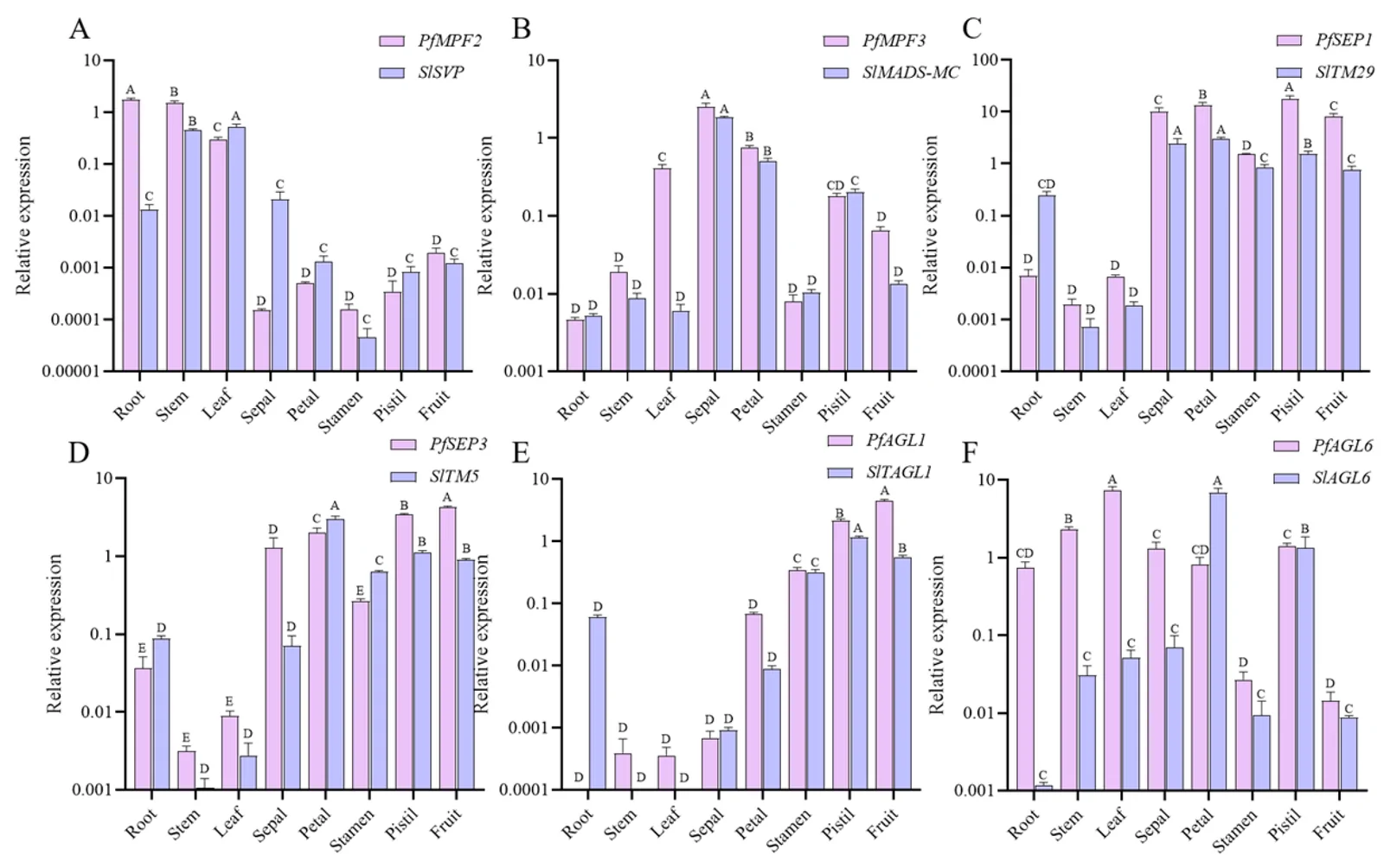
The expression patterns in different organs of genes related to sepal development in *P. pubescens* and *S. lycopersicum*. (**A**): PfMPF2, MADX box gene 2 from *P. pubescens*; SlSVP, Short Vegetative Phase; (**B**): PfMPF3, MADS box gene 3 from *P. pubescens*; SlMADS-MC, MACROCALYX; (**C**): PfSEP1, Physalis floridana SEPALLATA1; SlTM29, Tomato MADS box gene No.29; (**D**): PfSEP3, Physalis floridana SEPALLATA3; SlTM5, Tomato MADS box gene No.5; (**E**): PfAGL1, Physalis floridana AGAMOUS-like 1; SlTAGL1, Tomato AGAMOUS-like 1; (**F**): PfAGL6, Physalis floridana AGAMOUS-like 6; SlAGL6, Solanum lycopersicum AGAMOUS-like 6. The capital letters indicate statistical significance at *p* < 0.01 as determined by Duncan’s test.

To investigate the molecular regulation of sepal development, we analyzed the expression patterns of six *MADS-box* genes related to sepal development in different organs. *PfMPF2* (*MADS box gene 2* from *P. pubescens*) was predominantly expressed in vegetative organs of *P. pubescens*, especially in roots, whereas its *S. lycopersicum* ortholog *SlSVP* (*Short Vegetative Phase*) was mainly expressed in stems and leaves. *PfMPF3* (*MADS box gene 3* from *P. pubescens*), *PfSEP1* (*Physalis floridana SEPALLATA1*), and *PfSEP3* (*Physalis floridana SEPALLATA3*) were highly expressed in floral organs of *P. pubescens*, while their *S. lycopersicum* orthologs *SlMADS-MC* (*MACROCALYX*), *SlTM29* (*Tomato MADS box gene No.29*), and *SlTM5* (*Tomato MADS box gene No.5*) showed distinct organ specific expression patterns. *PfAGL1* (*Physalis floridana AGAMOUS-like 1*) and its ortholog *SlTAGL1* (*Tomato AGAMOUS-like 1*) were both specifically expressed in pistils and fruits. *PfAGL6* (*Physalis floridana AGAMOUS-like 6*) was expressed in vegetative organs, whereas *SlAGL6* (*S. lycopersicum AGAMOUS-like 6*) was expressed in floral organs of *S. lycopersicum* (Figure 10). These results indicate that the organ specific expression of these *MADS-box* genes has substantially diverged between the two species, which may underlie their distinct sepal developmental programs.

**Figure 11:**
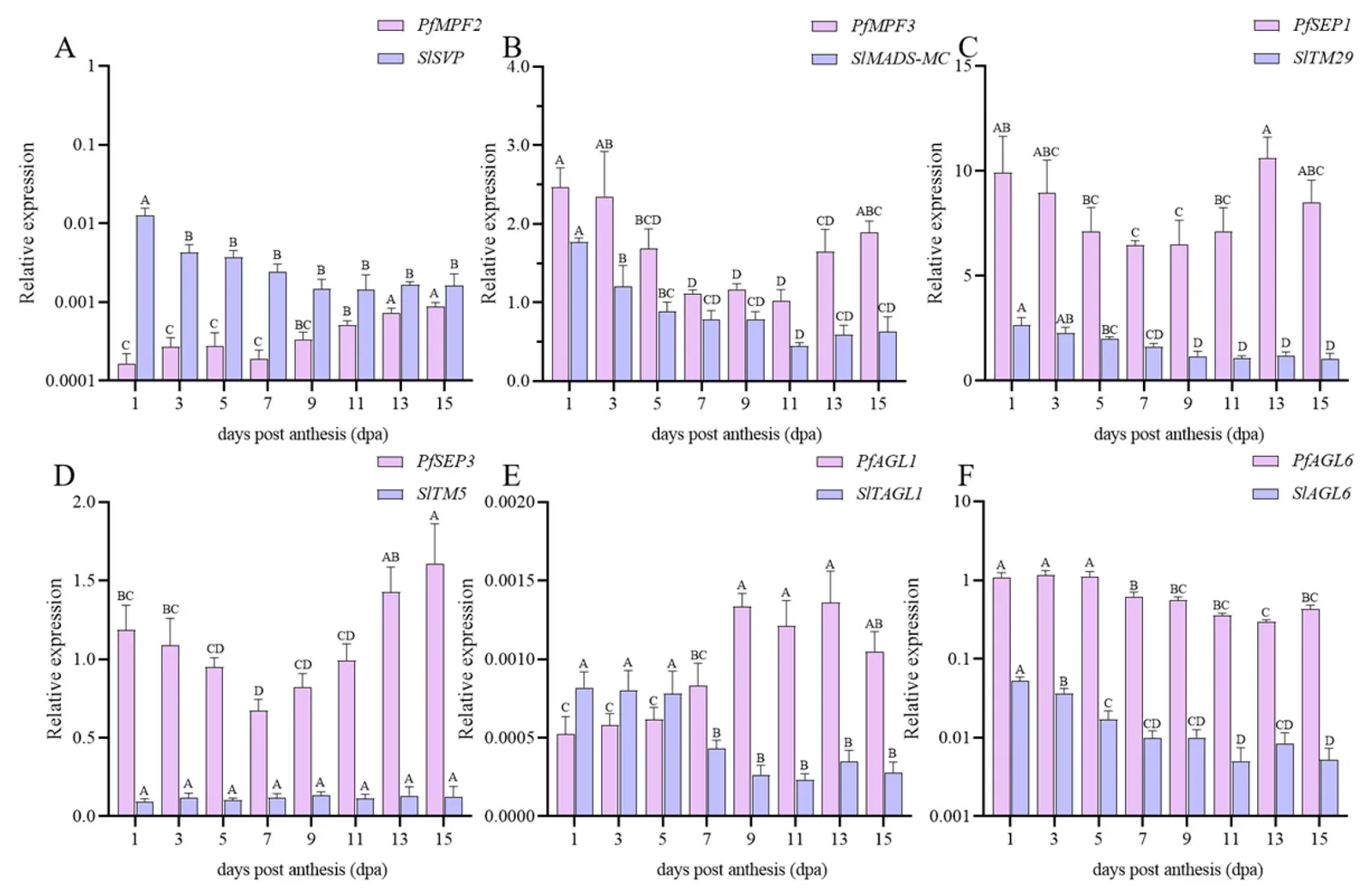
The expression patterns in different sepal development stages of genes related to sepal development in *P. pubescens* and *S. lycopersicum*. (**A**): *PfMPF2*, *MADX box gene 2* from *P. pubescens*; *SlSVP, Short Vegetative Phase*; (**B**): *PfMPF3*, *MADS box gene 3* from *P. pubescens*; *SlMADS-MC*, *MACROCALYX*; (**C**): *PfSEP1*, *Physalis floridana SEPALLATA1*; *SlTM29*, *Tomato MADS box gene No.29*; (**D**): *PfSEP3*, *Physalis floridana SEPALLATA3*; *SlTM5*, *Tomato MADS box gene No.5*; (**E**): *PfAGL1*, *Physalis floridana AGAMOUS-like 1*; *SlTAGL1*, *Tomato AGAMOUS-like 1*; (**F**): *PfAGL6*, *Physalis floridana AGAMOUS-like 6*; *SlAGL6*, *Solanum lycopersicum AGAMOUS-like 6.* The capital letters indicate statistical significance at *p* < 0.01, as determined by Duncan’s test.

Throughout sepal development, these genes exhibited divergent temporal expression dynamics between the two species. *PfMPF2* expression continuously increased during sepal development, while *SlSVP* declined in *S. lycopersicum* sepals. *PfMPF3*, *PfSEP1*, and *PfSEP3* displayed a high–low–high expression pattern, with expression minima at the period of fastest sepal growth (7 dpa) suggesting the negative regulatory roles; the orthologs genes of *S. lycopersicum* showed distinct expression patterns. *PfAGL1* was significantly upregulated after 7 dpa, coinciding with rapid sepal expansion, whereas *SlTAGL1* declined over the same period. The *PfAGL6* and *SlAGL6* both declined progressively during sepal development. These results indicate that the accelerated sepal growth in *P. pubescens* is temporally associated with the upregulation of positive regulators (*PfMPF2*, *PfAGL1*) and the transient downregulation of negative regulators (*PfMPF3*, *PfSEP1*, *PfSEP3*), while *PfAGL6* showed a progressive decline, a pattern temporally associated with the later stages of sepal development (Figure 11).

## 3. Discussion

### 3.1. Phylogenetic Framework for Divergent Reproductive Development

The phylogenetic analysis consistently placed *P. pubescens* in a well-supported clade with *P. alkekengi* and *P. ixocarpa*, clearly separated from the tomato subgroup. This is consistent with previous molecular phylogenies of the *Solanaceae* [24,25] and provides an evolutionary context for the divergent reproductive traits we observed. The substantial genetic distance between *P. pubescens* and tomato supports the interpretation that their regulatory networks controlling reproductive development have undergone significant divergence since their last common ancestor. This phylogenetic separation emphasizes the value of comparative studies between these two genera for understanding how morphological novelties arise during evolution.

### 3.2. Cellular Basis of Inflated Sepal Formation in P. pubescens

Our comparative morphological and microstructural analyses demonstrated that the dramatic post-anthesis expansion of the *P. pubescens* sepal is driven jointly by the marked enlargement of epidermal cells (particularly the inner epidermis) and the gradual formation of intercellular spaces within the parenchyma, eventually giving rise to a hollow lantern-like structure by 15 dpa. This is consistent with the established view that the early growth of the *P. pubescens* sepal depends on *PfMPF2*-driven cell division [20]. However, our data further reveal that the subsequent rapid expansion phase relies primarily on cell vacuolization and the development of intercellular spaces. Rapid organ expansion typically requires cell wall loosening through the action of expansions and xyloglucan endotransglucosylase/hydrolases (XTHs) [26], whose expression and activity are under precise developmental and hormonal control [27]. The coordinated up- and down-regulation of MADS-box genes observed in this study may therefore directly or indirectly modulate these cell wall-related processes to drive the phase-specific growth of the lantern structure. Nevertheless, the specific regulatory mechanisms underlying these processes in the *Physalis pubescens* sepal remain to be clarified.

The inner epidermis of the *P. pubescens* sepal is completely devoid of trichomes, in sharp contrast to that of *S. lycopersicum*. Trichome initiation in plants is regulated by the MYB-bHLH-WD40 (MBW) transcriptional complex [28,29]. The polarized distribution of trichomes, with their absence on the inner epidermis of the *Physalis pubescens* sepal, may result from asymmetric regulation of this complex between the two epidermal layers. Whether this absence of trichomes relieves physical constraints on the outward expansion of inner epidermal cells, thereby promoting the inward curvature of the lantern, represents a morphological question worthy of further investigation.

### 3.3. Divergent Microspore Development and Anther Morphogenesis

Our detailed analysis of microspore development revealed a previously undocumented temporal divergence between these two solanaceous species. *P. pubescens* was delayed relative to *S. lycopersicum* before the tetrad stage but accelerated after tetrad release, with pollen maturation and shedding occurring slightly earlier. This pattern suggests evolutionary plasticity in the relative timing of early versus late microspore developmental phases. However, the most striking cellular difference was the dramatic proliferation of connective tissue between locules in tomato anthers, which progressively expanded to occupy more than half of the locular volume at anthesis. This feature was completely absent in *P. pubescens*. Connective tissue proliferation has been noted in other *Solanum* species [30] and may influence pollen dispersal dynamics, but its developmental regulation and functional significance remain poorly understood. The absence of this trait in *P. pubescens* identifies it as a derived character within the *S. lycopersicum* lineage and provides a unique opportunity for comparative functional genomic studies.

### 3.4. Dynamic Regulation of Sepal Growth by MADS-Box Genes

The expression analysis of six *MADS-box* genes revealed a striking temporal correlation between gene expression patterns and sepal growth dynamics. *PfMPF2* expression continuously increased during sepal development, consistent with its established role as a master positive regulator of inflated sepal syndrome [4]. *PfAGL1* was significantly upregulated after 7 dpa, precisely coinciding with the onset of rapid sepal expansion. Given that overexpression of *SlTAGL1* (the tomato ortholog) can convert tomato sepals into fruit-like, expanded organs [9,31], the coincident upregulation of *PfMPF2* and *PfAGL1* suggests cooperative function, potentially through multimeric complexes bridged by SEPALLATA proteins to promote cell division and expansion.

The high–low–high expression pattern of *PfMPF3*, *PfSEP1*, and *PfSEP3*, with expression minima precisely at 7 dpa (the time of maximal growth rate), strongly connects these genes as growth repressors. *PfMPF3*, the A-class floral identity gene, is known to directly repress *PfMPF2* in floral meristems [19]. The parallel downregulation of *PfSEP1* and *PfSEP3* suggests that repression of inflated sepal syndrome involves a broader network. SEP proteins are essential cofactors in nearly all floral quartets [32], and recent studies have highlighted their role beyond organ identity maintenance, namely in modulating organ size and growth rate through differential complex formation [33]. Thus, reduced availability during the rapid growth phase may shift the balance of *MADS-box* complexes from organ identity maintenance toward growth promotion. In *S. lycopersicum*, downregulation of *SlTM29* leads to enlarged sepals [21], consistent with a growth-limiting role for *SEP* genes. The subsequent re-upregulation of *PfSEP1* and *PfSEP3* after 11 dpa coincides with growth deceleration and may serve as a feedback mechanism that prevents uncontrolled sepal expansion. *PfAGL6* expression declined continuously in both species. *SlAGL6* lineage genes control floral organ boundary formation and fusion [22,34], and the progressive decline in *PfAGL6* coincided with the completion of sepal expansion, suggesting a potential role in the terminal differentiation rather than directly promoting convergence. Whether *PfAGL6* downregulation contributes to the lantern shape through modulating organ boundary formation remains to be experimentally determined. Our findings thus establish a temporal framework in which the oscillating expression of SEP-class *MADS-box* genes tightly gates the growth-versus-differentiation balance during sepal inflation.

## 4. Materials and Methods

### 4.1. Plant Materials and Growth Conditions

The seeds of *P. pubescens* were obtained by cutting fresh fruit from Harbin and air drying. The seeds of *S. lycopersicum No.22* were stored at Shanghai Jiao Tong University, China. All plant lines were grown in a standard greenhouse at the Pujiang experimental base, Shanghai Jiao Tong University, Shanghai, China (121°30′10.89″ E, 31°3′5.20″ N, altitude 5 m).

### 4.2. Phylogenetic Analysis

Genomic DNA was extracted from young leaves of *P. pubescens*, tomato, pepper, eggplant and *Arabidopsis* by CTAB method [35], and PCR amplification and sequencing were carried out with corresponding primers described previously [36,37]. According to the sequencing results, phylogenetic tree was constructed by using MEGA7.0, neighbor joining (NJ) and 1000 repeated Bootstrap tests [38].

### 4.3. Botanical Characteristics Analysis

The *P. pubescens* and *S. lycopersicum* buds were taken every two days from 0 dpa to 17 dpa, sepal was peeled and photographed, and the length and width of sepal was measured with vernier caliper. The *P. pubescens* and *S. lycopersicum* buds were photographed every other day from −11 dpa to 0 dpa and fixed in FAA liquid (Sangon, Shanghai, China) for microspore development research. Photographs of the *P. pubescens* and *S. lycopersicum* fruits were taken every 4 days after flowering.

### 4.4. Pollen Vitality Analysis

Stamens of *P. pubescens* and *S. lycopersicum* were collected, and pollen was pricked with tweezers to take pollen on the glass slide and dropped 2–3 drops of 1% magenta acetocarmine staining solution. The pollen was incubated at 37 °C for 20 min to stain the pollen [39], and observed it under the microscope (Eclipse80i, Nikon, Tokyo, Japan). Fertile pollen grains were scored as those with round morphology and deep staining; sterile grains were scored as shrunken and weakly stained or unstained. At least 3 fields/20 grains were counted per sample.

### 4.5. Light Microscopy (LM) of Semi-Thin Sections

The *P. pubescens* and *S. lycopersicum* buds and sepals (cut into 3 × 2 mm^2^) at different development stages were immediately immersed in FAA fixed solution, and then stored in fresh FAA fixative at 4 °C [40]. The samples were dehydrated through a graded ethanol series (70%, 80%, 95%) for 30 min each, followed by 100% ethanol for 2 h. Dehydration was continued in a saturated solution of eosin in absolute ethanol for 2 h, after which the samples were infiltrated in a pre-infiltration solution (absolute ethanol: Basne Luqizd Technoovt 7100 = 1:1, *v*/*v*) for 2 h. The specimens were then placed in infiltration solution (1 g Hardener I dissolved in 100 mL Basne Luqizd Technoovt 7100) overnight at 25 °C. The samples were embedded in the embedding solution (hardener II: osmotic solution = *v*:*v* = 1:14) in an ice bath, heated at 48 °C for 10 min, and hardened in an oven at 60 °C for 48 h. Semi-thin sections (3 μm thick) were cut with a microtome (RM2265, LEICA, Wetzlar, Germany), stained with 1% toluidine blue O (TBO) (Sangon, Shanghai, China) for 40 s, rinsed with double-distilled water to remove excess stain, dried, and mounted with neutral balsam [41]. The sections were observed and photographed under a microscope (Eclipse 80i, Nikon, Tokyo, Japan).

### 4.6. Scanning Electron Microscopy (SEM)

The *P. pubescens* and *S. lycopersicum* sepals (cut into 3 × 2 mm^2^) at different development stages were immediately immersed in 2.5% glutaraldehyde (25% glutaraldehyde: 0.1 M phosphate buffer: double distilled water (*v*:*v*:*v*) = 1:5:4, pH = 7.3) for fixing. The samples were then placed under vacuum using a vacuum drying cabinet with a vacuum pump for 0.5 h, and then left at 4 °C for 12 h [42]. The samples were rinsed three times (for 15 min each) using 0.1 M phosphate buffer (pH = 7.2), immersed in 2% (*w*/*v*) osmium tetroxide for further fixing for 2 h, washed three times using 0.1 M phosphate buffer (15 min each time), and then dehydrated in an ethanol and acetone series as follows: 50% ethanol, 70% ethanol, 90% ethanol, 90% ethanol and acetone (*v*:*v* = 1:1), 90% acetone, each step for 15 min. Finally, the samples were dehydrated in acetone with shaking at 100 rpm (three times, 20 min each). After dehydration, the samples were immersed in acetone and epoxy resin 812 in different ratios (*v*:*v* = 1:1 and 1:2, for 1 h and overnight, respectively). The samples were then positioned on an embedding plate with 100% epoxy resin 812 for 7 h and kept at 60 °C for 48 h to form resin blocks. Thick sections (70 nm) were cut from the blocks using a Leica UC6-FC6 microtome with a diamond knife (Leica Microsystems Inc., Wetzlar, Germany) and stained with 2% lead citrate (*w*:*v* = 2%; Zhongjingkeyi Technology Co., Ltd., Beijing, China). The chromoplast ultrastructure was photographed using a scanning electron microscope (SEM; Verios G4, FEI, Hillsboro, OR, USA) at an accelerating voltage of 5–10 kV. Stomata and trichomes were identified and marked (St and Tr, respectively) on the SEM images.

### 4.7. Real-Time Quantitative PCR (RT-qPCR) Analysis

The sepals were sampled from *P. pubescens* and *S. lycopersicum* of the different stages for total RNA extraction using RNAprep pure Plant Kit (Tiangen, Beijing, China). Total RNA (1 μg) was used as a template to synthesize first-strand cDNA with the PrimeScriptTM RT Master Mix kit (TAKARA, Dalian, China) [43]. The cDNA library was diluted 50 times and used as a template for RT-qPCR analysis. RT-qPCR was performed on a Real-Time PCR System (QuantStudio 5, Thermo Fisher Scientific, Waltham, MA, USA) using SYBR Green chemistry (TAKARA, Dalian, China). The gene expression levels were calculated using the 2^−ΔCT^ equation with *ACTIN* as an internal reference gene for normalization. RT-qPCR primers are listed in Supplementary Table S1.

### 4.8. Statistical Analysis

All experiments were performed with at least three biological replicates. Data are expressed as mean ± standard deviation (SD). Statistical analysis was performed using SPSS 19. Differences between two groups were analyzed by Duncan’s multiple range test. Different letters indicate significant differences at *p* < 0.05 or *p* < 0.01.

## 5. Conclusions

In conclusion, this study presents analysis of the reproductive biology of *P. pubescens* and *S. lycopersicum*. Furthermore, we discovered that the inner epidermis of *P. pubescens* sepals was completely devoid of trichomes, in contrast to that of *S. lycopersicum*, which represented a novel morphological divergence potentially facilitating lantern formation by reducing physical constraints on inner epidermal cell expansion. Our results showed that the inflated sepal syndrome is driven by coordinated inner epidermal cell expansion, parenchyma intercellular space formation, and the dynamic regulation of a *MADS-box* gene network, with *PfMPF2* and *PfAGL1* acting as positive regulators and *PfMPF3*, *PfSEP1*, and *PfSEP3* as negative regulators. We further identified significant differences in microspore development and anther morphogenesis, most notably the pronounced connective tissue proliferation in tomato anthers that is absent in *P. pubescens*. These findings provide a cellular and molecular framework and reveal key divergences in reproductive development within the *Solanaceae*. From an applied perspective, the genes and developmental modules identified in this study may serve as targets for breeding programs aimed at modifying fruit protection traits in solanaceous crops. The trichome-free inner epidermis of *P. pubescens* sepals represents a selectable morphological marker, and the MADS-box regulatory network controlling sepal inflation could be exploited to engineer physical barriers against pests and environmental stress in related species.

## Figures and Tables

**Table 1:** The cell size of sepal epidermis of *P. pubescens* and *S. lycopersicum* (μm^2^).

dpa	(*P. pubescens*)		(*S. lycopersicum*)	
	Outer Epidermis	Inner Epidermis	Outer Epidermis	Inner Epidermis
1	287 ± 59	500 ± 73	789 ± 162	365 ± 64
3	350 ± 68	500 ± 69	855 ± 112	712 ± 103
5	339 ± 70	499 ± 39	859 ± 140	622 ± 160
7	374 ± 38	518 ± 83	814 ± 129	659 ± 44
9	935 ± 101	3560 ± 238	1483 ± 63	736 ± 143
11	2249 ± 395	3508 ± 682	1609 ± 293	646 ± 116
13	2486 ± 303	3441 ± 707	1716 ± 282	730 ± 165
15	2607 ± 413	3838 ± 600	2067 ± 150	902 ± 160

## Data Availability

The original contributions presented in this study are included in the article/Supplementary Material. Further inquiries can be directed to the corresponding author.

## Supplementary Materials

The following supporting information can be downloaded at: https://www.mdpi.com/article/10.3390/plants15162524/s1, Figure S1: Amplification of the target DNA fragment by PCR; Figure S2: The pollen fertility of *P. pubescens* and *S. lycopersicum*; Table S1: Primers used in this study.
