# Supplementary material for: Comparative Biology Research on the Reproductive Organs Between Physalis pubescens L. and Solanum lycopersicum var. cerasiforme
Source: Plants (Basel). 2026 Aug 20;15(16):2524. doi: 10.3390/plants15162524 (PMC13516136; doi:10.3390/plants15162524)
Supplement: Supplementary file 1 [file plants-15-02524-s001.zip › plants-4458798-supplementary.pdf]

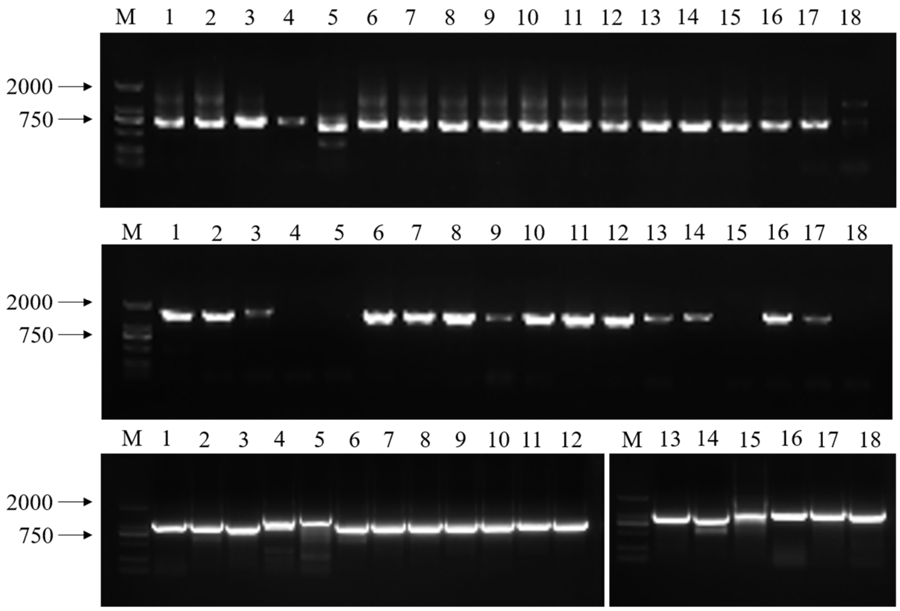

Supplementary Data figure S1

Amplification of the target DNA fragment by PCR

M: Marker DL 2000; 1: LA1585 (*S. pimpinellifolium*); 2: cv.P86 (*S. lycopersicum*); 3: Tobacco (*Nicotiana benthamiana*); 4: *Arabidopsis thaliana* (Columbia); 5: Downy groundcherry (*P. pubescens*); 6: Cherry tomato (*S.lycopersicum* var. *cerasiforme*); 7: LA2075 (*S. neorickkii*); 8: LA1042 (*S. cheesmaniae*); 9: LA1028 (*S. chmielewskii*); 10: LA1589 (*S. pimpinellifolium*); 11: LA2695 (*S. chmielewskii*); 12: LA0247 (*S. neorickkii*); 13: Black nightshade (*Solanum nigrum*); 14: Petunia (*Petunia hybrida*); 15: Tomatillo (*P. ixocarpa*); 16: Wintercherry (*P. alkekengi*); 17: Pepper (*Capsicum annuum*); 18: Eggplant (*S. melongena*).

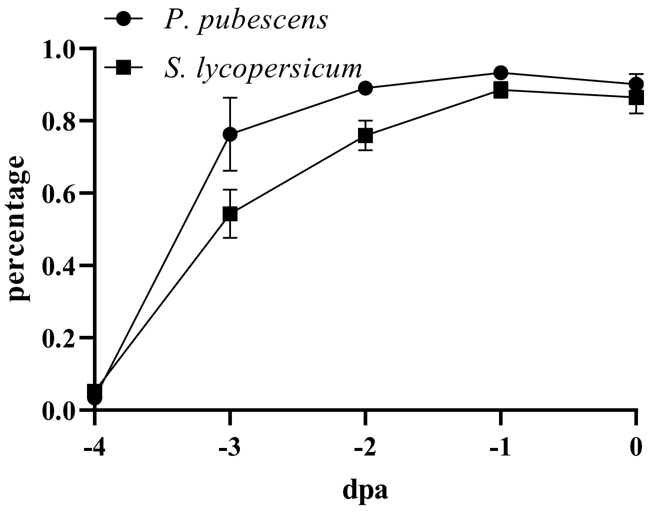

Supplementary Data figure S2

The pollen fertility of *P. pubescens* and *S. lycopersicum*

Supplementary Data Table S1 Primers used in this study

Primer name	Primer sequence (5′-3′)	Application
MPF2-F	gttctctgtgatgctgatgttgc	
MPF2-R	tcaagttcctcacctttcatctgtc	
SVP-F	gcaggtagctgataaaactcgtgag	
SVP-R	catcaccatgtcagtcactaaaggc	
MPF3-F	gatagtagggcaacaaggtgaatgg	
MPF3-R	ccatttctccttgcttcatctcctg	
MADS-MC-F	gatagtagaacagcaaggtgaatggc	
MADS-MC-R	cattattcctccttgcttctgctac	
Pf-SEP1-F	gcttatgaactatctgtgctttgtgatg	
Pf-SEP1-R	ccaactcatctccaagaaggtgtc	
TM29-F	gcttatgaactatctgtgctttgtgatg	
TM29-R	ccaactcatctccaagaaggtgtc	
Pf-SEP3-F	tgaaacaaaggttgatggaaggaagc	qRT-PCR
Pf-SEP3-R	ccagccagccatgtagttattcacac	
TM5-F	tgaaacaaaggttgatggaaggaagc	
TM5-R	ccagccagccatgtagttattcacac	
PfAGL1-F	gtacaagaaacaccatgctgattccac	
PfAGL1-R	ctccaactatttgcctgttataagtctg	
TAGL1-F	gtacaagaaacaccatgctgattccac	
TAGL1-R	ctccaactatttgcctgttataagtctg	
PfAGL6-F	agaacttcaacgaactcaaaggcac	
PfAGL6-R	caagatgacgctcctttctacgg	
SlAGL6-F	agaacttcaacgaactcaaaggcac	
SlAGL6-R	caagatgacgctcctttctacgg	
Actin-F	ttgctgaccgtatgagcaag	
Actin-R	ggacaatggatggaccagac	
